# Informatic challenges and advances in illuminating the druggable proteome

**DOI:** 10.1016/j.drudis.2024.103894

**Published:** 2024-01-22

**Authors:** Rahil Taujale, Nathan Gravel, Zhongliang Zhou, Wayland Yeung, Krystof Kochut, Natarajan Kannan

**Affiliations:** 1 Department of Biochemistry and Molecular Biology, University of Georgia, Athens, GA, USA; 2 Institute of Bioinformatics, University of Georgia, Athens, GA, USA; 3 School of Computing, University of Georgia, Athens, GA, USA

**Keywords:** protein evolution, orthology, network biology, machine learning, sequence embedding

## Abstract

The understudied members of the druggable proteomes offer promising prospects for drug discovery efforts. While large-scale initiatives have generated valuable functional information on understudied members of the druggable gene families, translating this information into actionable knowledge for drug discovery requires specialized informatics tools and resources. Here, we review the unique informatics challenges and advances in annotating understudied members of the druggable proteome. We demonstrate the application of statistical evolutionary inference tools, knowledge graph mining approaches, and protein language models in illuminating understudied protein kinases, pseudokinases, and ion channels.

## Introduction

Protein kinases and ion channels are among the most prominent families of druggable proteins. They play fundamental roles in cellular functions and disease,^(p1),(p2),(p3),(p4)^ and abnormal expression, mutation, or mis-regulation of these proteins is causally associated with various human disorders.^(p5),(p6),(p7),(p8)^ However, despite the biomedical importance of these proteins, a significant portion of the human protein kinases and ion channels remains understudied and is referred to as ‘dark’ by the Illuminating Druggable Genome (IDG) consortium.^(p9),(p10)^ The IDG Data and Resource Generation Centers (DRGCs) have generated valuable datasets, reagents, and chemical probes to illuminate understudied kinases, G protein-coupled receptors (GPCRs), and ion channels. Likewise, RNA interference (RNAi) screens of 260 conserved understudied genes have been performed in *Drosophila*, resulting in the generation of the ‘Unknome’ database.^(p11),^ The International Mouse Phenotype consortium has also generated thousands of mutant lines, which can be leveraged to identify disease associations for understudied genes.^(p12)^ While these and other ‘omic’ efforts have resulted in large volumes of data, effectively mining these data to annotate understudied proteins is a critical first step in identifying new drug targets. In particular, the wealth of sequence data available on protein kinases and ion channels from diverse organisms, combined with recent progress in structure prediction methods such as AlphaFold2,^(p13)^ provides valuable information for predicting and prioritizing understudied targets for drug discovery efforts. Likewise, integrative mining of sequence data in the context of cellular pathways, protein–protein interactions, cell type-specific expression, and disease mutations can identify novel disease associations for understudied members of the protein kinase and ion channel superfamily.^(p14)^

**FIGURE 1:**
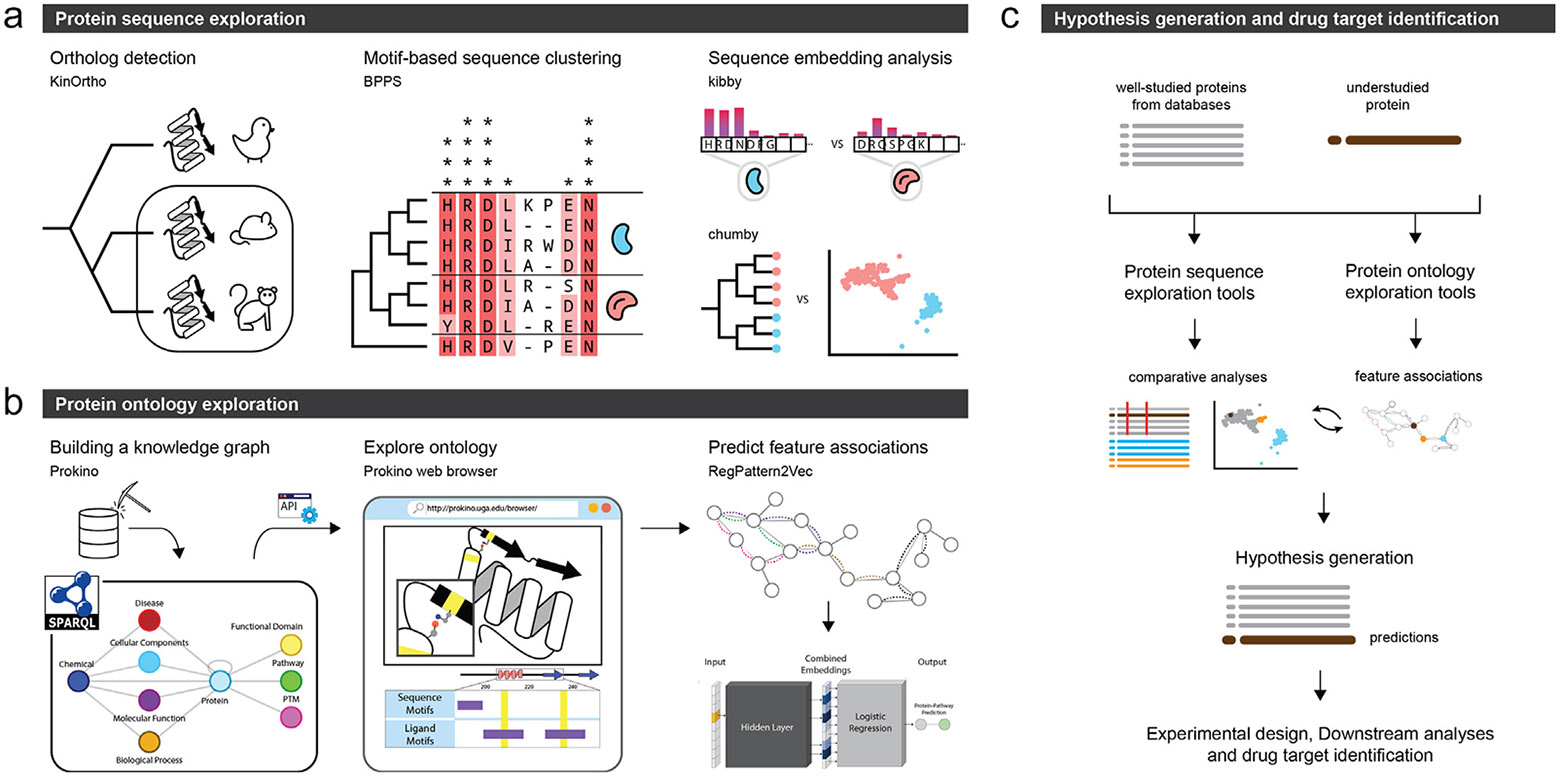
The panels represent the informatics approaches used for illuminating the druggable proteome. **(a)** Protein sequence exploration tools that enable analysis based on large collections of sequences, such as detection and collection of orthologs (KinOrtho), motif-based clustering of related sequences (BPPS), and embedding based-analyses (kibby and chumby). **(b)** Protein ontology exploration tools that rely on visualizing and querying prebuilt knowledge graphs to infer feature associations and predict functional and regulatory roles. **(c)** A schematic showing how the tools in panels (a) and (b) can be used iteratively to compare and contrast an understudied protein sequence with its well-studied counterparts to derive novel hypotheses and identify drug targets.

This review highlights the informatics challenges and advances in illuminating understudied proteomes. We focus on three related but complementary approaches (Figure 1). We first describe the application of orthology-based inference tools and associated statistical methods for predicting understudied protein functions based on the evolutionary context (Figure 1a, left). Next, we describe the development and application of knowledge graph (KG) mining approaches for function prediction using the network context encoded in KGs (Figure 1b). Finally, we review recent advances in sequence embeddings generated from protein language models for annotating understudied protein kinases and ion channels (Figure 1a, right). Figure 1c provides an overview of how these tools can be combined to derive novel hypotheses for understudied proteins and aid drug discovery efforts. A list of tools, and links thereto, are also provided in Table 1 for easy access. The abbreviations used in this review are listed in Table 2 for ease of reference.

## Orthology-based inference tools to illuminate understudied proteomes

The understudied protein kinases and ion channels belong to a large superfamily of evolutionarily related proteins, several of which are functionally well illuminated. Thus quantitative comparisons of primary sequences and three-dimensional structures can provide critical evolutionary clues for predicting the functions of understudied members of the protein kinase and ion channel superfamily. Indeed, sequence and evolution-based approaches have proven crucial in the prioritization and functional characterization of understudied kinases^(p15),(p16),(p17),(p18),(p19),(p20)^ and pseudokinases.^(p16),(p18),(p19),(p21),(p22)^ However, the lack of dedicated tools to map orthologs across species, and the challenges in accurately aligning divergent sequences, present a significant bottleneck in the sequence-based annotation of understudied protein kinases and ion channels.

Protein sequence data have been successfully employed to predict novel drug targets using methods such as prioritization based on gene essentiality,^(p23)^ identification of common targets across multiple pathogens,^(p24)^ or using a subtractive genomics approach.^(p25)^ Given the widespread popularity of these methods, several tools have emerged to identify orthologs, albeit with slight variations in how they define computationally defined orthologs. Most methods rely on an initial all *vs* all pairwise protein similarity search to identify reciprocal best hits, and then on phylogenetic, matrix-based, or distance-based clustering methods to define orthologous gene sets.^(p26),(p27),(p28),(p29),(p30),(p31),(p32),(p33),(p34),(p35),(p36),(p37)^ Resources such as ECOdrug^(p38)^ further enhance evolutionary conservation-based drug discovery by curating drugs and their protein targets across different species. As a common theme across these comparative genomics approaches, they traditionally begin by identifying and comparing evolutionarily related sequences across diverse organisms using multiple sequence alignment methods. For example, the KinVieW visualization tools enables consistent and reproducible evolutionary comparisons using a consistent set of curated alignments spanning protein kinase families from diverse organisms.^(p17)^ Likewise, statistical tools such as Bayesian partitioning with pattern selection (BPPS)^(p39)^ group evolutionarily related sequences based on the patterns of conservation and variation in large multiple-sequence alignments. In so doing, they enable the functional annotation of understudied members by identifying patterns shared among understudied and illuminated members of a protein superfamily. Indeed, these statistical sequence classification methods and visualization tools have been successfully employed for predicting and experimentally testing the functions of several understudied kinases, such as Unc-51-like kinase 4 (ULK4) and the dark pseudokinase, serine/threonine-protein kinase H2 (PSKH2).^(p15),(p18),(p19),(p22)^

More recently, an orthology mapping tool, KinOrtho, was developed to alleviate the challenge of leveraging evolutionary data for protein kinase function prediction.^(p20)^ KinOrtho employs a combination of query- and graph-based techniques, utilizing full-length and domain-based strategies to accurately map one-to-one kinase orthologs across an extensive set of 17 000 species. The effectiveness of KinOrtho is demonstrated through rigorous metrics, showcasing its superior performance compared to existing methods. KinOrtho enhances accuracy by identifying potential false positives and emphasizing sequences lacking proper kinase domains for further scrutiny, thus possessing great utility in dark kinase illumination. For example, KinOrtho detected a domain fusion event between the understudied TAOK1 and a functionally well-studied PIK3C2A kinase in a subset of nine nematode species. Subsequent coexpression analysis revealed a strong association between the two kinases extending well beyond nematodes to other organisms. This suggests a possible physical interaction between the two proteins and a functional association involving communication between the cell membrane and the cytosol.

Further, machine learning models have been leveraged to use the KinOrtho-defined orthologs for well-studied kinases, their sequence similarities, and manually curated Gene Ontology (GO) annotations as training features to derive functional predictions for the understudied kinases. This framework has predicted biological functions for various understudied kinases.^(p20),(p40)^ Moreover, depending on the model’s ability to assign putative functions, a quantitative score, called the Novel Inferred Annotation Score (NIAS) score, is also assigned to each understudied kinase, which prioritizes them for functional studies. Thus, KinOrtho is a valuable tool for hypothesis generation using evolutionary information encoded in protein sequences and can be extended to any druggable protein family of interest. This framework is being adopted to perform an orthology-based analysis of the human ion channelome. A similar study of full-length ion channel sequences and their isolated pore domains could shed light on the conserved functional associations of understudied ion channel sequences across diverse species. Identifying associated binding partners or tandem domains could be of interest for directing drug design efforts to provide fine-tuned control over channel functions and their underlying disease mechanisms.

## Data integration and knowledge graph mining approaches for function prediction and target identification

Data related to protein kinases and ion channels are stored in disparate data sources and formats, posing significant data integration and mining challenges. Thus, harmonized data resources linking related data on druggable gene families can be valuable for hypothesis generation and testing.^(p10)^ For example, Target Central Resource Database (TCRD)^(p41)^ and Pharos^(p14)^ are centralized resources with several built-in tools for users to identify and prioritize new targets for functional illumination or target discovery. These resources offer user-friendly GraphQL application programming interface (API) for enrichment analysis and tools for data visualization. Importantly, these resources provide an annotation that classifies targets based on their available data, known as the target development level (TDL), allowing researchers to prioritize understudied proteins for drug discovery efforts. Likewise, DrugCentral^(p42)^ aggregates data on new drug approvals and standardizes drug information, including preclinical research and clinical practice data such as chemical structures, molecular physicochemical descriptors, and patent status. Multiple additional resources have been developed that provide information on understudied targets’ structural^(p43)^ and pathway contexts. Of note is the Reactome knowledgebase, a curated knowledgebase of biological pathways successfully employed for predicting the biological functions and therapeutic potential of understudied dark proteins.^(p44)^ Likewise, the dark kinase knowledgebase^(p40)^ and LinkedOmics database^(p45)^ capture additional experimental, proteomic, and disease-related data on understudied dark proteins.

While aggregated databases and harmonized resources serve as a valuable starting point for target identification, identifying hidden patterns in harmonized data resources requires data minability. KGs are a powerful solution to data representation and integration challenges. They semantically link disparate data sources into a structured resource description format (RDF), enabling efficient data sharing, storage, and mining.^(p46),(p47),(p48),(p49)^ One such example is the Protein Kinase Ontology (ProKinO), which integrates data related to sequence, structure, function, pathway, gene expression, and ligand binding sites on protein kinases^(p50),(p51)^ in human- and machine-readable formats. Both graph mining techniques and machine learning on ProKinO data have enabled the illumination of understudied kinases using network context. For example, ProKinO mining using the SPARQL protocol and RDF query language (SPARQL) identified p21-activated protein kinase 5 (PAK5) as a frequently mutated dark kinase in human cancers, including a previously unrecognized role in acute myeloid leukemia.^(p51)^ Furthermore, the impact of oncogenic mutations on PAK5 structure and function was predicted using the structural visualization tools built into the ProKinO browser.^(p51)^ In another example, effective integration of informatics and experimental approaches resulted in the functional illumination of the understudied pseudokinase, PSKH2. Specifically, quantitative comparisons of PSKH2 orthologs from diverse organisms identified primate-specific ‘pseudogenization’ of PSKH2 based on variations in the active site. Subsequent analysis of AlphaFold2 models and cell-based assays established the role of the N and C-terminal segments flanking the pseudokinase domain in cellular localization and interaction with the Hsp90 molecular chaperone.^(p22)^ Furthermore, mining of ProKinO KG using the link prediction algorithm, RegPattern2Vec, predicted multiple pathways for PSKH2 including a role for PSKH2 in cilium assembly.^(p52)^

**FIGURE 2:**
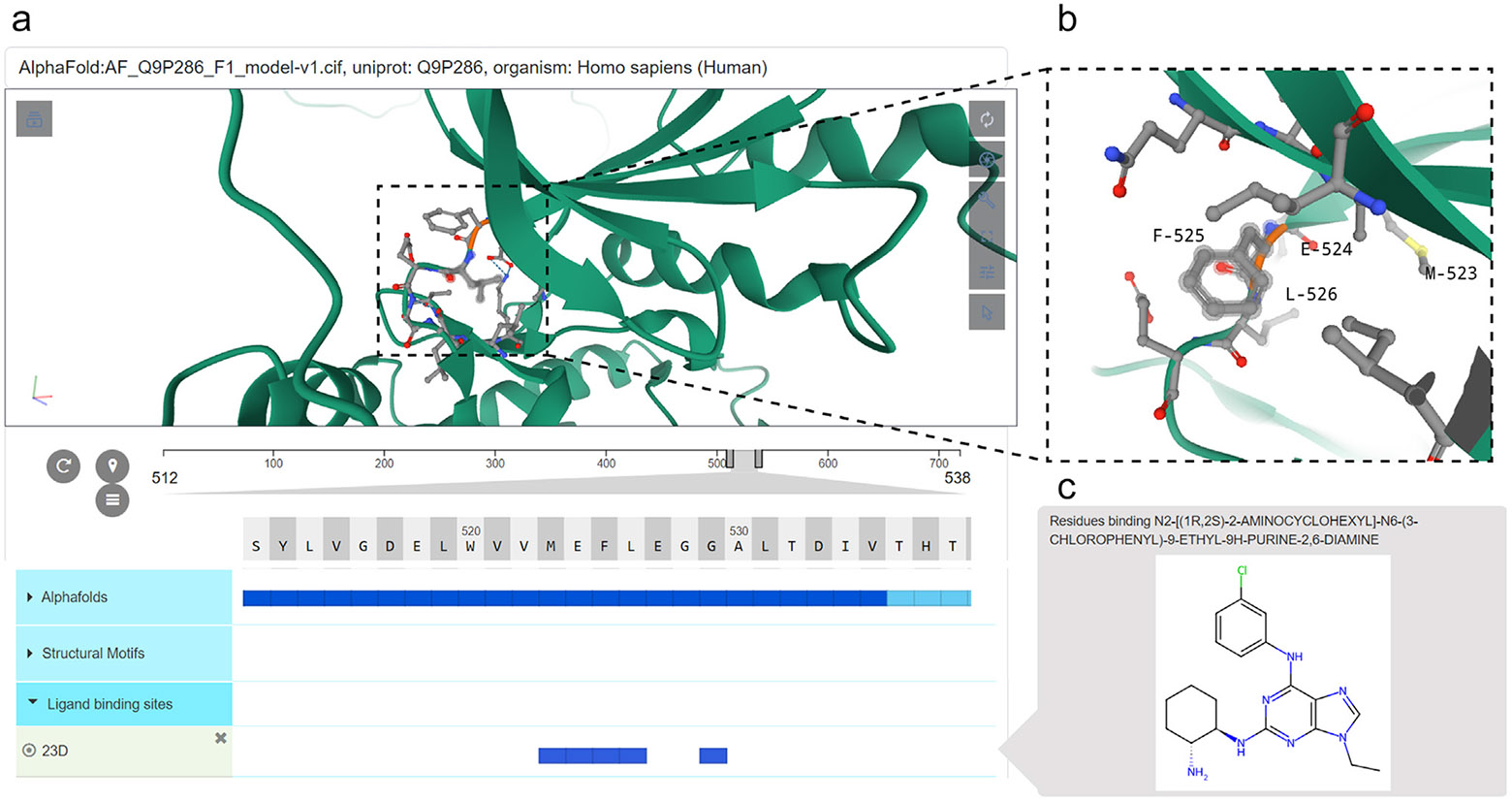
A demonstration of the ProtVista viewer within the ProKinO browser for mapping sequence annotations to 3D models using the p21 activated kinase 5 (PAK5) as an example. **(a)** Snapshot of the ProtVista viewer showing key sequence annotations within the ligand binding site of PAK5. **(b)** Zoomed-in view of the ATP/ligand binding residues. **(c)** Schematic of a small molecule targeting the active site of PAK5.

More recently, the addition of drug (ligand) classes and their relationships to protein kinase structural features using semantic relationships (edges) with structural motifs has enabled queries and hypothesis generation regarding the mode of binding of kinase drugs within the ATP or allosteric pockets (Figure 2), making ProKinO a valuable tool for drug discovery efforts.

In addition to knowledge discovery through graph mining, KGs can be leveraged for machine learning tasks. In particular, graph representation learning through network embedding approaches^(p53)^ offers the possibility of identifying hidden patterns or relationships (links) in large heterogeneous datasets. Indeed, various groups and IDG data management centers have successfully employed machine learning on KGs for predicting relationships between protein kinase inhibition and cancer;^(p54),(p55)^ placing kinases in a pathway context;^(p52)^ and predicting kinase substrates,^(p56),(p57)^ drug and disease associations,^(p58),(p59)^ and COVID-19 responses;^(p49)^ among other applications.^(p60),(p61),(p62)^ A significant challenge in the practical application of machine learning on KGs is the development of vector representations that effectively capture both local and latent contexts encoded in KGs. Various approaches for graph representation learning have been proposed,^(p63)^ including the random walk-based method, RegPattern2Vec, which utilizes regular pattern-constrained random walks to capture multiple aspects of the node context within the KG.^(p52),(p64)^ Learning functional representations from a kinase-centric KG establishes contextual information for kinases, interacting partners, modifications, pathways, localization, and chemical interactions. These known representations are then used to predict pathway associations for understudied kinases. Using RegPattern2Vec, Salcedo *et al.* predicted pathway associations for 30 dark kinases,^(p52)^ and the predictions aligned well with pathway enrichment data obtained from experimentally generated protein proximity networks.^(p40)^ A unique aspect of the RegPattern2Vec approach is that it enables the biological interpretation of the predictions by listing the collected random walks used in vector representations, thereby enabling the functional annotation of understudied targets.

## Language models for illuminating druggable proteomes

Recent advances in deep learning open the exciting possibility of predicting understudied protein functions using protein language models trained on millions of sequences cataloged in sequence databases.^(p65)^ These models represent inferred structural and functional properties of proteins as vector embeddings, capturing hidden patterns and nuances that traditional alignment-based methods may otherwise miss. Sequences represented as vectors, or sequence embeddings, can be leveraged for various function annotation tasks, such as predicting post-translational modifications,^(p66)^ protein–protein interactions,^(p67)^ protein–drug interactions,^(p68)^ evolutionary conservation,^(p69),(p70)^ and structure.^(p71),(p72)^ Lin *et al*., for example, recently employed ESMFold, a fully end-to-end single-sequence structure predictor that relies on the ESM2 transformer language model, to internalize encoded evolutionary information and perform single-sequence structure predictions with high speed and accuracy. This task constitutes an alternative to AlphaFold2 structure-prediction^(p73)^ by eschewing the need for a traditional sequence alignment, thereby making structure prediction robust even against proteins with minimal sequence representation (orphan proteins).

Alongside structure prediction, sequence embeddings generated from language models have also been successfully employed for alignment-free conservation estimation^(p69),(p74),(p75)^ in three druggable protein families. These data, along with other protein families, can be visualized using the sequence annotation viewer in Pharos.^(p14)^ These embeddings have also been successfully employed for alignment-independent embedding-based protein classification,^(p70)^ protein structure prediction,^(p71),(p72),(p76)^ homology detection, structural alignment,^(p77),(p78)^ and predicting kinase–substrate associations.^(p79)^ These advancements have significant implications for illuminating understudied ion channels, which have been challenging to study using traditional alignment-based approaches because of the extensive diversification of ion channel sequences and 3D structures. Many ion channels share little homology in sequence space, with the difficulties in structural characterization being a critical barrier to understanding ion channel function.^(p80)^ Both issues hinder structure prediction approaches like AlphaFold2, which depend on accurate sequence alignments and experimentally derived models for training.^(p81)^ Therefore, using sequence embeddings to capitalize on inferred structure–function properties from a protein language model may help overcome the challenges associated with alignment-based approaches.

**FIGURE 3:**
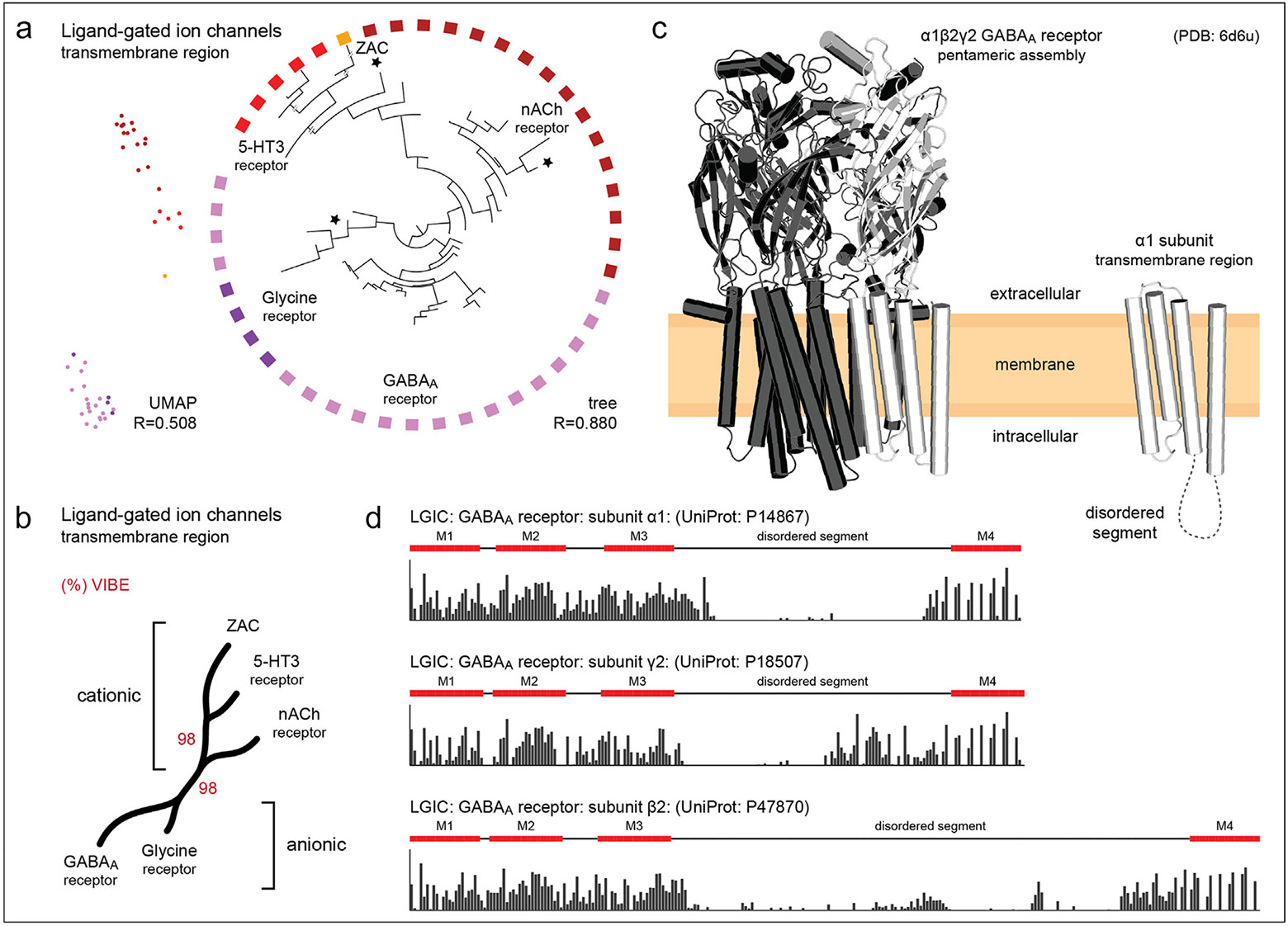
**(a)** An embedding tree of the human Cys-loop ligand-gated ion channels using the transmembrane region. Nodes with a black star indicate the dark ion channels in this subfamily. To the left of the tree, we plot a UMAP projection using the same dataset. **(b)** A condensed tree showing various families of Cys-loop ligand-gated ion channels. The red percentage values indicate VIBE scores. **(c)** Cryo-electron microscopy structures of an example membrane-bound ligand-gated ion channel, human α1β2γ2 GABA_A_ receptor.^(p86)^ The structural model on the left shows the heteropentameric assembly built from three unique subunits: α1, β2, and γ2. Each monomer consists of an N-terminal extracellular domain, which adopts a β-sandwich, followed by the C-terminal transmembrane domain. Within the pentamer, an α1 subunit is colored white. The structure on the right depicts the transmembrane domain of an α1 subunit. **(d)** Embedding-based sequence conservation for the transmembrane region of GABA_A_ receptor subunits α1, β2, and γ2. Each consists of four transmembrane helices, designated M1–4, and a disordered segment on the M3–4 loop. Bars indicate the level of conservation at that sequence position, with taller bars indicating higher conservation.

As an illustration of the successful application of such a strategy, recent efforts focused on employing a sequence embedding-based classification and visualization method (chumby)^(p70)^ to delineate the distant evolutionary relationships within the human Cys-loop ligand-gated ion channel families. An embedding-based tree (Figure 3a) was successfully generated using the conserved transmembrane segments within these sequences. Consistent with previous functional classifications,^(p82),(p83)^ the embedding based classification clearly separates the cationic receptors [5-hydroxytryptamine type 3 (5-HT3), nicotinic acetylcholine (nACh), zinc-activated channel (ZAC)] from anionic receptors [glycine, γ-aminobutyric acid type a (GABA_A_)] (Figure 3b), with 98 % statistical support estimated by the Variational Autoencoder Implemented Branch Support Estimation (VIBE) scores.^(p70)^ This separation is also observed in the Uniform Manifold Approximation and Projection (UMAP) plot (Figure 3a). The glycine receptor family contains one dark ion channel sequence [gamma-aminobutyric acid receptor subunit pi (GABRP)]. In contrast, the nACh family has two dark ion channel sequences [acetylcholine receptor subunit beta (CHRNB1) and neuronal acetylcholine receptor subunit alpha-10 (CHRNA10)] grouped with other Cys-loop family members (stars in Figure 3a).

Interestingly, CHRNA10 is placed closest and basal to the 5-HT3 and ZAC families. In the cellular context, the Cys-loop ligand-gated receptor superfamily members are known to form homo- or heteropentameric assemblies. For instance, the human α1β2γ2 GABA_A_ receptor is a pentameric assembly of three unique subunits: α1, β2, and γ2 (Figure 3c). Additionally, we estimate sequence conservation across these three subunits using sequence embeddings^(p69)^ which reveal highly conserved regions corresponding to the four conserved transmembrane helices. Low conservation scores indicate fast-evolving regions (Figure 3d), such as those observed between the M3 and M4 helices, which reflect variable functions among Cys-loop members such as receptor modulation, sorting, and trafficking. In contrast, the high conservation observed in the transmembrane domain reflects shared functions, such as multimeric assembly and gating mechanisms.^(p84)^ Thus, such alignment-free identification of fast- and slow-evolving sites can be employed for uncovering functionally important regions of members of the dark channel subfamily (GABRP, CHRNB1, CHRNA10).

## Concluding remarks

The IDG consortium has generated valuable datasets and resources to characterize understudied protein kinases, ion channels, and GPCRs. The next challenge is translating these data and resources into knowledge for drug discovery efforts. Here, we highlight the informatics challenges and advances in annotating understudied proteomes. Focusing on understudied protein kinases and ion channels, we demonstrate the application of evolutionary inference, KG mining, and protein language models in the functional annotation of understudied members of these protein families. In particular, sequence representations generated from protein language models can be leveraged for the functional annotation of understudied ion channels, which have been challenging to study using traditional alignment-based approaches. Likewise, fine-tuning of large language models with experimentally derived substrate-specific profiles of kinases generated from peptide-library studies^(p85)^ holds excellent promise in mapping the phosphorylation networks of the entire kinome and placing understudied kinases in a pathway context. Finally, approaches integrating KGs and protein language models can be powerful new tools for translating genomic discoveries into therapeutic strategies.

## Figures and Tables

**TABLE 1:** List of informatics tools for illuminating understudied proteins

Tool name	Description and location
KinOrtho	Full-length and domain-based orthology detection https://github.com/esbgkannan/KinOrtho
KinVieW	Visualization tool for correlating natural sequence variation with cancer variants and post-translational modifications in the protein kinase domain https://prokino.uga.edu/kinview/
BPPS	Bayesian pattern-based hierarchical sequence partitioning https://www.igs.umaryland.edu/labs/neuwald/software/bpps/
ProKinO	An integrated knowledge graph linking diverse forms of data on protein kinases https://prokino.uga.edu/
RegPattern2Vec	A random walk-based graph embedding approach for link prediction tasks https://github.com/esbgkannan/RegPattern2Vec
chumby	Sequence embedding-based tree visualization https://github.com/esbgkannan/chumby
kibby	Alignment free embedding-based sequence conservation analysis https://github.com/esbgkannan/kibby
Pharos	Knowledge base for the Druggable Genome to illuminate understudied or poorly characterized portions of protein families https://pharos.nih.gov/

**TABLE 2:** Abbreviations and their full forms provided for reference in the order they appear in the review

Abbreviations	Referring to
IDG	Illuminating Druggable Genome
DRGCs	IDG Data and Resource Generation Centers
GPCRs	G protein-coupled receptors
RNAi	RNA interference
BPPS	Bayesian partitioning with pattern selection
ULK4	Unc-51-like kinase 4
PSKH2	Serine/threonine-protein kinase H2
TAOK1	Thousand and one amino acid protein kinase 1
PIK3C2A	Phosphatidylinositol 4-phosphate 3-kinase C2 domain-containing subunit alpha
GO	Gene ontology
NIAS	Novel Inferred Annotation Score
TCRD	Target Central Resource Database
TDL	Target development level
KGs	Knowledge graphs
RDF	Resource description format
ProKinO	Protein Kinase Ontology
SPARQL	SPARQL Protocol and RDF Query Language
PAK5	p21-activated protein kinase 5
Hsp90	Heat shock protein 90
ATP	Adenosine triphosphate
ESM2	Evolutionary Scale Modeling 2
VIBE	Variational Autoencoder Implemented Branch Support Estimation
UMAP	Uniform Manifold Approximation and Projection
GABRP	Gamma-aminobutyric acid receptor subunit pi
CHRNB1	Acetylcholine receptor subunit beta
CHRNA10	Neuronal acetylcholine receptor subunit alpha-10
5-HT3	5-Hydroxytryptamine type 3
ZAC	Zinc-activated channel
GABA	Gamma-aminobutyric acid
nACh	Nicotinic acetylcholine

## Data Availability

Data will be made available on request.
